# A comprehensive environmental scanning and strategic analysis of Iranian Public Hospitals: a prospective approach

**DOI:** 10.1186/s13104-020-05002-8

**Published:** 2020-03-26

**Authors:** Kimia Pourmohammadi, Peivand Bastani, Payam Shojaei, Nahid Hatam, Asiyeh Salehi

**Affiliations:** 1 Health Care Management, Health Human Resources Research Center, School of Management and Medical Informatics, Shiraz University of Medical Sciences, Shiraz, Iran; 2 Health Services Management, Health Human Resources Research Center, School of Management and Medical Informatics, Shiraz University of Medical Sciences, Shiraz, Iran; 3 Department of Management, Shiraz University, Shiraz, Iran; 4 School of Health and Human Sciences, Southern Cross University, Gold Coast, Queensland Australia

**Keywords:** Public hospital, Environmental scanning, Strategic management, PESTLE analysis, Future, Iran

## Abstract

**Objectives:**

This study was conducted to provide a strategic direction to public hospitals in Iran via environmental scanning in order to equip hospitals to plan and perform proactively and adapt with the everchanging environment.

**Results:**

A mixed method study including in-depth interview and survey were used to determine influential environmental factors based on PESTLE (political, economic, social, technological, legal and environmental) and Douglas West framework to determine the effectiveness and feasibility of factors. Issues identified at micro environmental level were over prescription, inequality in distribution of healthcare services and high demands for luxurious health services. Issues identified at the macro environmental level were related to changes in disease patterns, inappropriate hospital budgeting, economic sanctions, government corruption and healthcare centralization. In order to tackle the issues identified, it is paramount to enhance bed distribution management, improve strategic policies for a more equitable payment system, and enhance the efficiency and effectiveness of services by implementing a strategic inventory control. Furthermore, the considerable impact of economic sanctions on financial resources of Iranian hospitals should not be ignored.

## Introduction

Organizations’ environments are changing at an unprecedented rate [1], posing substantial effect on healthcare systems [2–4]. As healthcare systems, play a major role in social and financial development and welfare, lack of awareness of the environmental change, can result in severe health-related complications for the population health [2, 5–7]. Hospitals have a major role in the fairness index in healthcare [2, 5–7]. They are the most fundamental and expensive components of the health system, using 40% and 80% of total health sector expenses in developed and developing countries, respectively [8–11].

Environmental changes result in political, economic, social, cultural, and technological changes at organizational levels, such as hospitals. Some of the key changes are population aging, health technological advances, information technology developments, and remote medical systems [12]. Healthcare organizations need to adapt with this rapid environmental changes to assure the sustainability of their services [2, 13].

Environmental scanning acts as a radar for identifying environmental signals, and help with developing compatible strategies to direct the organization in the adaptable way [14]. Hence, it is an effective strategic process, in this complicated uncertain healthcare system [15]. Environmental scanning predict and comprehend internal and external organizational factors and their interconnectedness to decrease the level of uncertainty [16, 17]. For example, it identifies threats and opportunities that potentially affect performance or jeopardize the organizational sustainability or performance [13], to gain sustainable competitive advantages [14].

The organization environment consists of external and internal components. The external environment, include micro and macro environments is related to factors outside the normal borders of the organization affecting management decisions [18]. The macro environment includes factors with indirect long-term political, economic, social, cultural, technological, and legal impacts. While, the micro environment refers to factors that directly affect organizational functions and outcomes, such as customers, suppliers/resources, competition, and other stakeholders [14, 18]. This study aimed to identify the environmental factors affecting Iranian public hospitals (using a prospective approach) to provide a strategic direction for achieving high quality and at the same time, efficient services.

## Main text

### Methods

#### Study design

This mixed-method study was conducted in 2017–2018 in two phases:

##### Phase 1: Analyzing influential environmental factors in Iranian public hospitals

**Fig. 1:**
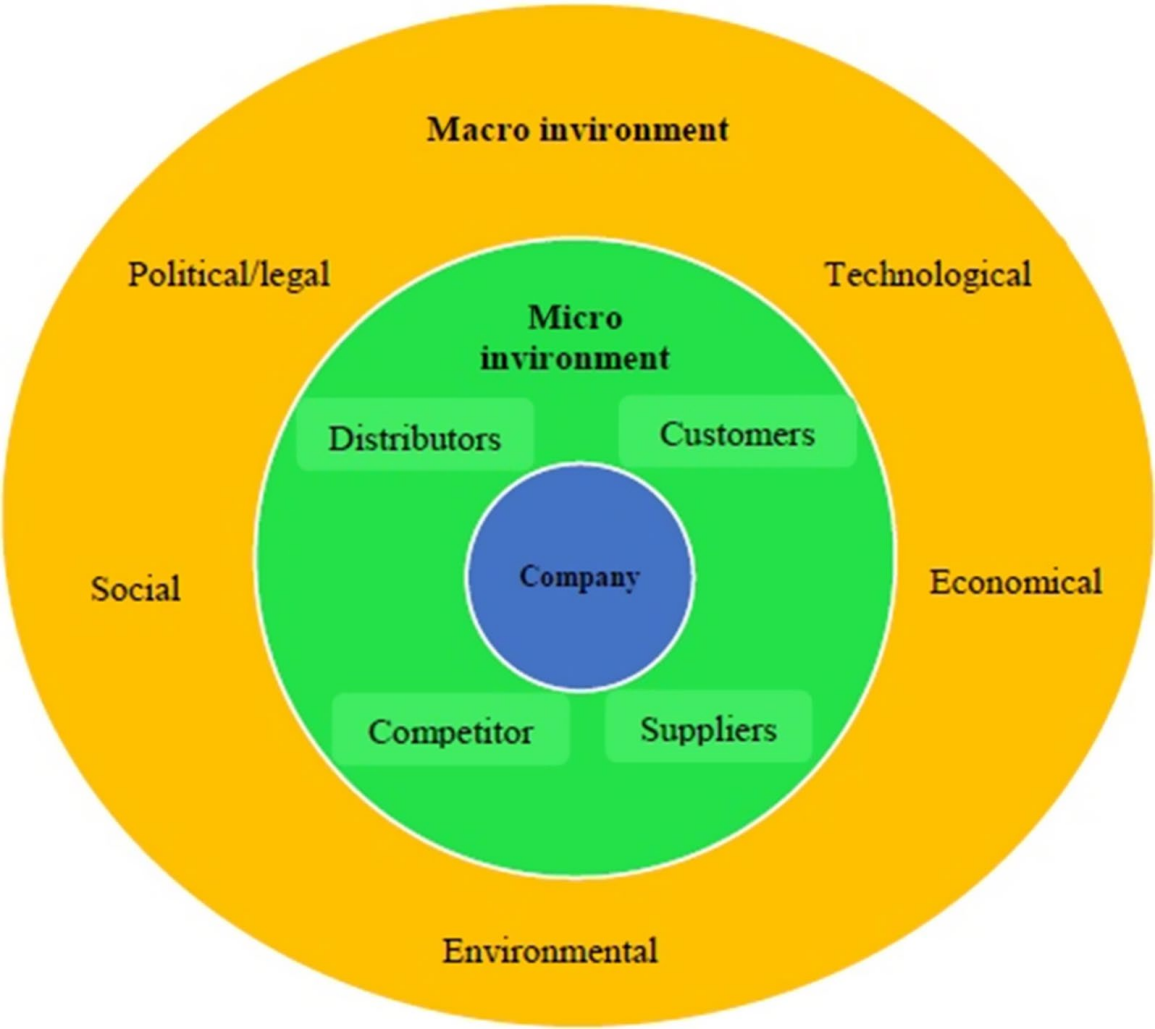
Organization environment analysis framework (West et al. [18])

In this phase, political, economic, social, technological, legal, and environmental factors influencing the macro situation of Iranian public hospitals were identified via PESTLE (political, economic, social, technological, legal and environmental) analysis. The micro environmental factors such as customer, public, media, distributors, suppliers, stakeholders, and competitors were further analyzed using the framework proposed by Douglas West et al. [18] in Fig. 1.

Semi-structured in-depth interviews were conducted among a panel of experts from diverse ranges of disciplines in healthcare to achieve a comprehensive understanding of the influential factors. Participants were informed about the research purpose. The initial sample size was estimated to be 12 experts. Considering possible withdrawal, 16 experts were selected. Individual interviews with 16 experts were saturated. Purposive snowball sampling was used for the selection of participants.

The questions were structured based on West et al. framework. Four panel sessions were held to finalize interview analyses (90-min). The participant consent was obtained to record and take notes of all interviews and panel meetings. Recorded files transcribed, and shared with the participants for verification and possible feedback.

Data analysis was performed using the deductive method, including familiarization, identifying a thematic framework, indexing, charting, and mapping and interpretation [19]. MAXQDA-11 was used for data analysis. A final expert panel session was also held in order to apply corrections based on their initial views to reach a consensus around the extracted factors and their relevance to research purposes.

##### Phase 2: Determining the impact and uncertainty of environmental factors impacting Iranian hospitals

At this stage, a researcher-made questionnaire based on first stage was used to determine the level of impact and uncertainty (via a 5-point Likert scale). The numbers [1–5] indicated the amount of influences that each factor had on hospital performance and (±) denoted opportunity and threat respectively. Experts (n: 32) were asked (via phone) for their permission to complete the questionnaires, afterwards, the questionnaire was sent electronically. Average views of the participants on every question were determined to analyze.

### Results

Influential environmental factors were divided into micro and macro environmental factors, which are both interconnected. The influential factors at the micro level categorized into (1) *consumers*, including socio-demographic and socio-economic status, health literacy, inclinations to use luxurious services, demands for receiving high-quality and/or modern technologies’ (2) *distributors,* such as not equitable distribution in hospital beds, staffing and pharmaceutical resources), (3) *stakeholders*, including *internal stakeholders* (clinical and nonclinical staff, faculty member, medical and nonmedical students, patients, carers and family members), *External stakeholders* (insurance companies, ministry of health and medical education, physicians, professional organization and nursing professional organization) and (4) *competitors* (home care and nursing care services). Table 1 indicates further details about the impacts of these factors on public hospitals. Macro environmental factors were classified as political, economic, social, technological, legal, and environmental dimensions. Table 2 indicates further details about the effectiveness and certainty of these factors.

**Table 1:** Environmental impact matrix (micro environment)

Dimensions	Factors	Impact of factors	Potential opportunities/threats
Customer, public and media	More inclination towards using luxurious health services	Increased costs, higher quality services, overuse of complicated expensive technologies	− 3
	More demand for high quality health services	Higher costs, human resources, expensive equipment	− 2
	Increase in average income	Increase in hospital income	+ 5
	Increase in purchasing power	Increase in hospital income	+ 5
	Education level and health literacy improvement	Decrease in hospitalization period and increase of bed turnover rate	+ 3
	Organic and green products attitude	Decrease in diseases and demand for health services which will lead to quality improvement in public hospitals	+ 3
	Environment protection and green energy use attitude	Increase in hospital expenses for healthy waste disposal and use of latest technologies with green energy	+ 1
	Increase in people’s share in health services payments	Increase in hospital specific income	+ 3
Distributors	Unfair bed distribution	Longer patient wait times leading to disorder and lower quality of services	− 4
	Unfair specialized human resources distribution	Longer patient wait times and non-responsiveness	− 5
Suppliers	Increase in prescription of drugs out of Iranian official list of drugs	Inability to supply drugs and lower quality of services	− 2
	Increase in the number of prescriptions containing antibiotics	Patients’ resistance to treatment and higher doses of drugs leading to medicine supply issues	− 2
	Increase in the number of prescriptions containing injections	Patients’ resistance to treatment and higher doses of drugs leading to medicine supply issues Increase in design costs and equipping hospitals with clean rooms	− 2
	Increase in the number of self-medication cases in patients	Increase in the number of patients with no appointments leading to longer patient wait times	− 3
Stakeholders	Delayed payment to hospitals by insurance companies	Hospitals being indebted and therefore unable to supply medicine and consumer products or purchasing low quality products that in turn will lead to patient dissatisfaction. In addition, delay in personnel reimbursement can result in lack of satisfaction and motivation to provide high quality care.	− 5
	Full-time status of clinical faculty members (non-permissibility of simultaneous work in both public and private sectors)	Shorter patient wait times and more responsiveness	+ 4
	Freedom of speech in media, multiplicity of political parties, civil rights, meetings or campaigns to support or ban health policies (social - political)	Compromised reputation of public hospitals due to myriad economic and political issues	− 2
Competitors	More inclination towards receiving home care and nursing care	Shorter wait times and improved quality of services and opportunities for launching home care	+ 3
	Growth in usage of health promotion software	Decreased rate of referring to hospitals and shorter wait times	+ 2
	Growth of clinics and private hospitals	Shorter wait times in public hospitals and improved quality of services	+ 5
	Important basic infrastructures (facilities and installations) in Iran and the city in which the hospital is located	Remote medical services and electronic medical record option	+ 5
	Good academic and knowledge developments in Iran and the city in which the hospital is located	Improved treatment processes and quality of services and patient satisfaction	+ 3
	A chance to make the required investments for research and development in Iran	Improved treatment processes and quality of services and patient satisfaction	+ 4
	Good developments in high-end technologies in hospitals	Improved treatment processes and quality of services and patient satisfaction	+ 3
	Availability of high-end technologies in the relative industry of hospitals	Higher hospital expenses	− 2
	Available required communication structures Good developments in information and communication technology	Remote medical services and electronic medical record option	+ 5
	Electronic commerce option for hospitals	Income generation Growth of medical tourism industry	+ 4
	Using social media to promote hospital products	Income generation	+ 4

**Table 2:** Environmental impact and certainty (Macro environment-PESTLE analysis)

Aspects	Factors	Influence	Certainty
Political	Regional competitions	− 1	− 3
	Policy makers’ neglect of the health sector	− 4	− 3
	Centralization in the dominant attitude	− 4	− 5
	Government budget-cutting structure	− 5	− 5
	Implementation of the Family Physician Program	+ 2	+ 3
	Periodic changes of politicians leading to change of plans of directors (political instability)	− 3	− 5
	Lack of appropriate philosophy and viewpoint about health and its various dimensions among political parties and formations	− 3	− 4
	Government downsizing based on various laws, including the 44th principle (privatization development)	+ 4	+ 3
	Government financial corruption	− 4	− 4
	Unreasonable tariffs determined for hospitals products and services	− 5	− 5
	Political sanctions	− 4	− 5
Economic	Improved payment system structure (strategic services purchase by insurance companies based on quality and price)	+ 5	+ 3
	Improved tariff structures	+ 4	+ 2
	Improved drugs and consumption products purchase control structure	+ 5	+ 3
	Higher inflation in the health sector	− 5	− 5
	Higher expenses (drugs and treatment)	− 5	− 5
	Higher inflation	− 4	− 5
	Higher bank interest rates	− 4	− 4
	Improved financing structure	+ 5	+ 3
	Currency rate fluctuations and multiplicity of currency rates	− 4	− 5
	Supportive role of government financial policies	+ 5	+ 2
	Providing access to capital/loans to develop hospitals’ activities by the government	+ 4	+ 4
	Good market economic growth	+ 3	+ 1
	Availability of required finances (from public government budget, charities, etc.) to produce hospitals products and services	+ 5	+ 2
	Smaller budget share for the health sector	− 5	− 5
	Approved national Iranian pharmacopoeia and the comprehensive list of equipment	+ 3	+ 3
	More budget limitations for the health sector as a result of economic and health load of non-communicable and emerging diseases because of environmental changes	− 3	− 3
	Economic sanctions	− 3	− 5
Social and cultural	Higher population growth	− 3	− 5
	Higher fertility rates	− 3	− 4
	Change of diseases load towards chronic illnesses	− 5	− 5
	Lower physical activity	− 3	− 4
	Higher life expectancy	− 3	− 4
	Higher poverty	− 4	− 5
	Appropriate population distribution (young human resources to total population ratio)	+ 2	+ 4
	Appropriate family size and structure	+ 2	+ 3
	Higher rates of social harms and anomalies, including divorce, crimes, and violence.	− 3	− 4
Technology	Improved health information technology (home care, remote medical services, remote training, electronic medical record)	+ 4	+ 2
Legal	Lack of legal clarity for hospitals activities development	− 4	− 4
	Tax and employment laws ratified by the government	− 4	− 4
	Inappropriate budgeting system for hospitals (general budget, linear budget, ownership of the remaining budget resulting from frugality)	− 5	− 4
	Deficiency in health technologies evaluation (import permits for high-end technologies and expensive drugs)	− 4	− 5
	Poor supportive laws for attracting domestic and international investors in manufacture, equipment, and renovation of hospitals (including bank laws, facilities, loans, letters of guarantee)	− 3	− 5
	The requirement for hospitals to observe scientific and local guidelines approved by the Ministry of Health and insurance companies	+ 5	+ 2
Environmental	Higher risks and diseases resulting from environment pollution	− 3	− 4
	Higher air pollution in cities in which the hospitals are located	− 4	− 5
	The possibility of unexpected events in the city where the hospitals are located	− 4	− 3
	Greater possibility of man-made disasters in the city where the hospitals are located	− 3	− 3
	Population positive attitude toward green energy	+ 3	+ 3
	Population positive attitude toward green and organic products	+ 3	+ 3

### Discussion

Findings indicated that the micro environmental factors affected the quality of services as well as their expenditures. One of the key micro factors is the lengthy waiting time (as indicated in Table 1), impacting the efficiency, effectiveness and customer satisfaction [20–23]. The results of a meta-analysis by Fazel Hashemi et al. showed that this indicator was higher in the emergency departments of Iranian hospitals in comparison with national and international standards. Another important aspect at this level is inequitable distribution of hospital beds, professional and pharmaceutical resources, reducing responsiveness and patient satisfaction. Therefore, it is substantial to revisit the resource distribution at different healthcare levels (e.g., prevention, education, and research and treatment sections) as well as managing the efficiency of resources based on referral system [7].

The macro environmental factors impact hospitals directly or indirectly. Factors with direct impact, include higher fertility rates, hospital services tariffs, changes in the patterns of diseases, and hospital budgeting. While, factors with indirect impact include stakeholders, distributors, economic sanctions, government corruption, centralization and high bank interest rates.

Health and illness are considered a social phenomenon [24], impacted by the aging population (up 20% of the Iranian population by 2050) as a direct macro factor [25]. This indicates changing the disease patterns, which require updated technologies to enhance the self-efficacy/self-control of individuals (e.g., incorporating advanced digital health an artificial intelligence in healthcare system). In addition, this requires increasing the community-based services, and involving patients and their family members/carer, in decision making about their health and the services to access to the right service at the right time [26, 27]. Other interconnected social determinants of health in Iranian society are related to unhealthy lifestyle behaviors, poverty, outskirts/assembly residential, drug abuse/addiction, lack of physical activities, which can result in more chronic illnesses and threatening public hospitals and their care provision to individuals [28].

From the *economic* point of view, public investments in the health system has not increased in proportion to the increased health costs [29–31]. One of the main issues that hospitals are confronted is the payment system (fee for services), which is designed to encourage service providers to offer more services [32]. Regarding the mega trend of change from volume-based to value-based paradigm [28], one of the innovative methods can be strategic service purchase or service package [32, 33].

Implementation of HTP (health transformation plan) is also a good strategy to decrease the out-of-pocket (OOP) payments for inpatient services and eradicate informal payments to physician. Furthermore, delegation of some costly parts of hospitals to private partners based on the “public–private-partnership (PPP)” models can be a beneficial solution for enhancing the harmony between Iranian health policies and change of paradigm from volume to value [33].

Downsizing was identified as one of the main factors in *political* dimension. This intervention can improve the performance of public hospitals by reducing bureaucratic costs, service delivery duration, increasing efficiency and enhancing skills [34]. In addition, as this study indicated, healthcare *technological* advances can be assessed and used appropriately to decrease the burden on healthcare system and enhance the efficiency of services. Some examples of usage of advanced technology can be related to home care services, remote medicine, remote training, electronic medical record and smart hospitals. Overall, it is required to localize technology and apply HTA (health technology assessments) to enhance the appropriate usage of health technologies based on the needs of patients and general population. Selecting the appropriate budgeting system for hospitals (contraction–expansion) was identified as the main factors in *legal* dimension due to increased economic and health burden of non-communicable diseases and newly-emerged diseases caused by environmental changes.

### Conclusion

Three key recommendations were provided to improve the quality and at the same time, efficiency of services, in Iranian hospitals and the healthcare system as a whole. First, it is required to revise the current referral system into a more sustainable one (e.g., decreasing the number of unnecessary referrals to specialists), to enhance the cost-efficiency and equitability of care, particularly in remote and rural areas. Secondly, small public hospitals need to be supported by some strategic plans, such as integration to other hospitals and/or creating hospital chains/networks to work in collaboration, for a more holistic care provision. However, it is paramount to prevent the healthcare provision bias due to lobbying between large hospitals and pharmaceutical companies. Third, an appropriate technology assessment process is required to prevent over usage of technologies (particularly around chronic illnesses) and subsequent financial burden it can impose on the healthcare and the society. Fourth, shifting the hospitals and health care system into more community-based and holistic care system to look at the health and wellbeing from different perspectives and not only the physical aspects of the health.

## Limitations

This study is a cross-sectional view of the changing health system in Iran and as the “Environmental scanning” is a dynamic method, this process requires an update every 3–4 years to match the ever-changing situation.

## Data Availability

The datasets generated and analysed during the current study are not publicly available due to the confidentiality of the interviews but are available from the corresponding author on reasonable request.

## Abbreviations

PESTLE: Political, economic, social, technological, legal and environmental
Douglas West et al. framework: Examines key aspects of marketing strategies such as customer, public, media, distributors, suppliers, stakeholders, and competitors combined with the presentation of a synthesis of recent thinking on the subject
MAXQDA: Is a software program designed for computer-assisted qualitative and mixed methods data, text and multimedia analysis in academic, scientific, and business institutions
HTA: Health technology assessments
HTP: Health transformation plan
OOP: Out-of-pocket
PPP: Public–private-partnership
